# Supplementary material for: Association Between the Renin–Angiotensin System and Ibrutinib-Related Cardiovascular Adverse Events: A Translational Cohort Study
Source: Biomedicines. 2025 Sep 6;13(9):2184. doi: 10.3390/biomedicines13092184 (PMC12467112; doi:10.3390/biomedicines13092184)
Supplement: Supplementary file 1 [file biomedicines-13-02184-s001.zip › biomedicines-3711238-supplementary.pdf]

Supplementary data

Parameters	Thresholds
Creatinine (µmol/L)	100
CRP (ng/mL)	3589.5
Galectin-3 (ng/mL)	8.65
Myeloperoxydase (ng/mL)	209.05
Renin (pg/mL)	1336.1
Aldosterone (pg/mL)	488.95
TNF-alpha (pg/mL)	85
IL-6 (pg/mL)	9.75
ACE-2 (ng/mL)	3.63
Troponin (pg/mL)	127.15
miR-9 (zmol/µL)	0.19
miR-199 (zmol/µL)	13.45
miR-22 (zmol/µL)	3.23
miR-99 (zmol/µL)	0.9
miR-150 (zmol/µL)	27.38
miR-328 (zmol/µL)	3.11

Table S1: Optimal Thresholds for Cohort Stratification Determined by Youden's Index. CRP: C reactive protein; TNF: tumor necrosis factor; IL: interleukin; ACE-2: angiotensin-converting enzyme 2.
